# Management of Grade 1 Open Distal Radius Fractures: A Survey of Practicing Surgeons

**DOI:** 10.7759/cureus.54982

**Published:** 2024-02-26

**Authors:** Andrew Megas, Christopher Healy, Nicholas Frane, John M Tarazi, Kate Nellans, David Galos

**Affiliations:** 1 Department of Orthopaedic Surgery, University of Rochester Medical Center, School of Medicine and Dentistry, Rochester, USA; 2 Department of Orthopaedic Surgery, The Center Orthopedic & Neurosurgical Care & Research, Bend, USA; 3 Department of Orthopaedics and Trauma, The Center for Orthopedic Research and Education (CORE) Institute, Phoenix, USA; 4 Department of Orthopaedic Surgery, Northwell Health, Huntington Hospital, Huntington, USA; 5 Department of Orthopaedic Surgery, Donald and Barbara Zucker School of Medicine at Hofstra/Northwell, Hempstead, USA; 6 Department of Orthopaedic Surgery, Northwell Health, Long Island Jewish Medical Center, New Hyde Park, USA; 7 Department of Orthopaedic Trauma, Nassau University Medical Center, East Meadow, USA

**Keywords:** trauma, hand, grade 1 open distal radius fractures, survey of practicing surgeons, surgical management

## Abstract

Introduction

Standard of care management for open fractures historically mandates emergent systemic antibiotic administration, followed by urgent irrigation and debridement in the operating room, regardless of injury severity. However, significant controversy exists regarding the specific implementation and importance of these commonly accepted guidelines. We aimed to define differences in the management of grade 1 open distal radius fractures.

Methods

An anonymous online survey was distributed to attending surgeon members of either the Orthopaedic Trauma Association (OTA) between January 2019 and April 2019 or the New York Society for Surgery of the Hand (NYSSH) in January 2019.

Results

A total of 68 attending surgeons responded to the survey. A total of 24 OTA members and 40 NYSSH members replied and were included in the study. Several factors influenced management in addition to the level of contamination. Of the surgeons, 68% stated that litigation was not a major factor of concern. When compared to surgeons who trained in trauma fellowships, more surgeons who trained in hand/upper extremity fellowships considered closed reduction alone as reasonable definitive treatment (when excluding antibiotic administration and debridement considerations, p = 0.024) and oral antibiotics as a supplement to IV antibiotics (p < 0.001). Of the surgeons, 90% would nonoperatively treat a patient who presented with a grade 1 open distal radius fracture greater than 72 hours after injury with stable and acceptable alignment on X-rays.

Conclusion

Some surgeons are willing to deviate from standard-of-care management protocols.

## Introduction

Standard of care management for open fractures historically mandates emergent systemic antibiotic administration, followed by urgent irrigation and debridement in the operating room, regardless of injury severity [1]. However, significant controversy exists regarding the specific implementation and importance of these commonly accepted guidelines.

Countless studies demonstrate the role of systemic antibiotics in reducing infection rate in open fractures; however, the bacterial coverage, number of agents, duration of treatment, and role of adjunctive therapies remain debatable. While the use of cephalosporins, pioneered by Patzakis et al. studying cephalothin, remains iconic, evidence for an optimal antibiotic regimen is not absolute [2,3]. Broader antibiotic coverage for more severe or contaminated injuries commonly includes newer-generation versions of antibiotics or combination therapy, but this practice lacks substantive support. Some evidence suggests broadened antibiotic coverage provides insignificant benefits, may be redundant, and may include significant risks such as renal failure (however the latter has been called into question) [4-7]. Although the role of early antibiotic administration is irrefutable, the optimal duration of antibiotic administration lacks clear evidence [5]. Antibiotic administration beyond 24 hours after irrigation and debridement for grade 1 fractures, even in critically injured patients, may even increase the probability of antibiotic-resistant infections, with no additional protection against sepsis, organ failure, or death [8,9]. While the necessity of antibiotics in the management of open fractures is convincing, nearly every aspect of its administration is controversial.

In a meta-analysis, Zhang et al. presented compelling evidence for nonoperative treatment of grade 1 open forearm fractures in pediatric patients; zero infections occurred in 127 fractures and only three patients eventually required operative treatment for loss of reduction of the fracture (all without treatment or evidence for related infection) [10]. With newer evidence questioning, if not rejecting, previously held norms of need for emergent and operative irrigation and debridement in clean-contaminated low-grade open fractures, one could reasonably question the norms as “standard of care.” Our study attempts to define differences in the management of grade 1 open distal radius fractures, stratified by surgeon training.

## Materials and methods

An anonymous online survey was written and distributed to attending surgeons who manage grade 1 open distal radius fractures. The survey was distributed to members of either the Orthopaedic Trauma Association (OTA) or the New York Society for Surgery of the Hand (NYSSH). The survey was designed to stratify surgeons based on type of training and setting of practice to determine variations in the management of grade 1 open distal radius fractures. The survey for the OTA members was available on the OTA website (https://ota.org/research/research-surveys) from January 2019 to April 2019. The survey for NYSSH members was circulated via email distribution in January 2019.

Statistical analysis was performed using the R statistical package (R Foundation for Statistical Computing, Vienna, Austria). Demographics were assessed descriptively, and associations were analyzed using Fisher’s exact test for categorical variables. Significant associations were considered at a p-value less than 0.05. Because this is a pilot study, a power analysis was not performed.

## Results

**Figure 1:**
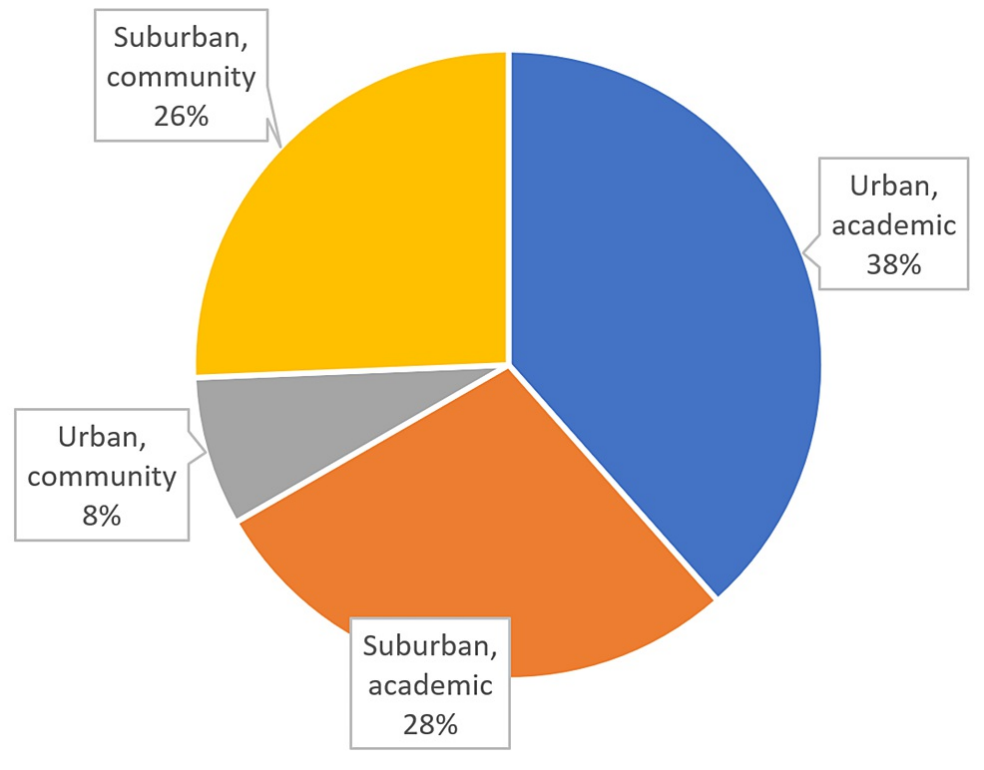
Survey respondents’ practice setting

Twenty-four of 770 targeted OTA members (response rate of 3.1%) and 40 of 110 NYSSH members (response rate of 36%) replied to the survey. Overall, 68 attending surgeons accounted for a combined response rate of 7.9%. Of the surgeons, 94% trained in an orthopedic surgery residency, while 4% trained in plastic surgery and 2% in general surgery. While most surgeons practiced in an urban and academic setting (38%), other surgeons practiced in suburban or community settings, with one respondent practicing in a rural community setting (Figure 1).

**Figure 2:**
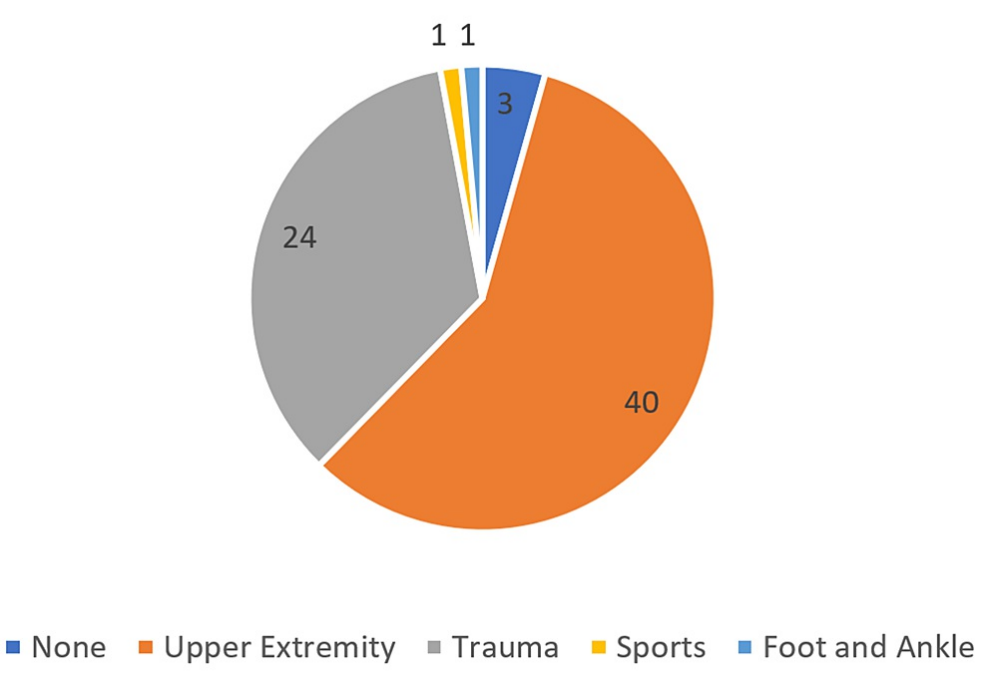
Survey respondents’ fellowship training

Of the surgeons, 58% had additional training in a hand/upper extremity surgery fellowship, and another 35% in an orthopedic trauma surgery fellowship (Figure 2). Only 4% of surgeons who responded did not have fellowship training (Figure 2).

**Figure 3:**
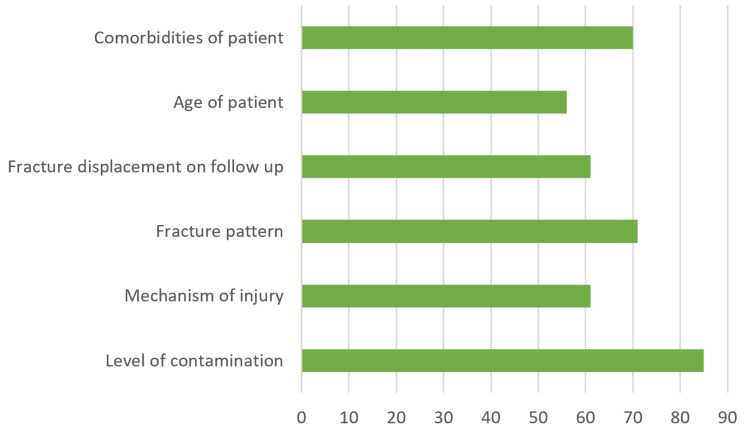
Contributing factors directing treatment protocol for grade 1 open distal radius fractures

Several factors influence the management, including the level of contamination, fracture pattern, displacement on follow-up, and patient comorbidities, according to responding surgeons (Figure 3).

**Figure 4:**
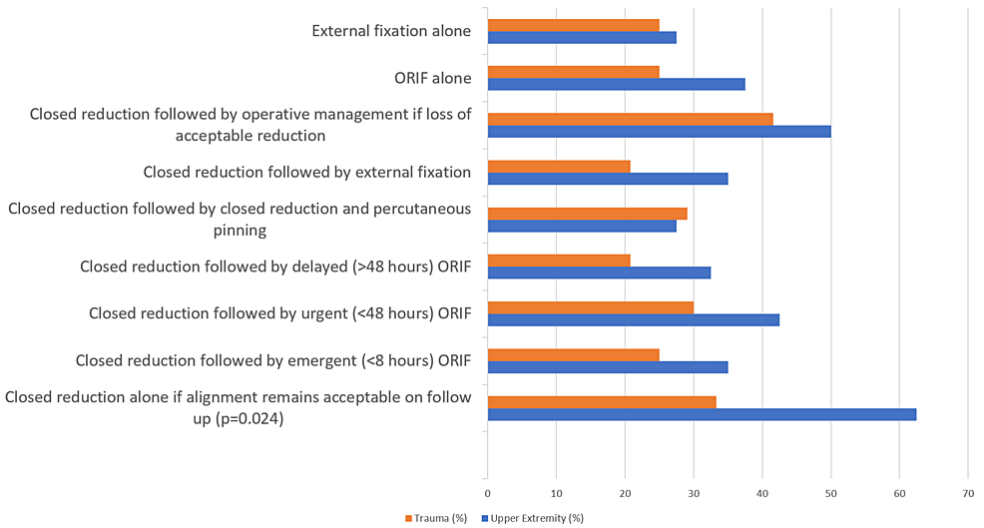
Potential reasonable treatment options for grade 1 open distal radius fractures, excluding considerations for antibiotic administration and debridement ORIF: open reduction and internal fixation.

Concerning definitive treatment (excluding antibiotic administration and debridement), there was a mix of what surgeons considered reasonable; however, significantly more surgeons who trained in hand/upper extremity fellowships considered “closed reduction alone if alignment remains acceptable on follow-up” when compared to surgeons who trained in trauma fellowships (p = 0.024, Figure 4).

**Figure 5:**
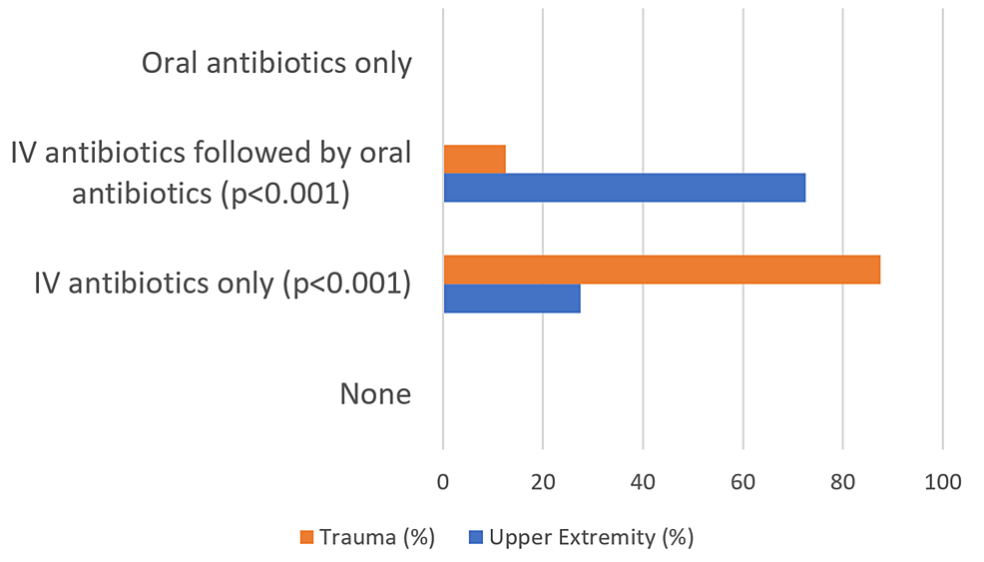
Surgeon preference for the route of antibiotic administration (oral or intravenous) for grade 1 open distal radius fractures at the time of injury

Furthermore, significantly more surgeons who trained in hand/upper extremity fellowships would prescribe IV antibiotics followed by oral antibiotics, whereas surgeons who trained in trauma fellowships overwhelmingly would prescribe only IV antibiotics (p < 0.001, Figure 5).

**Figure 6:**
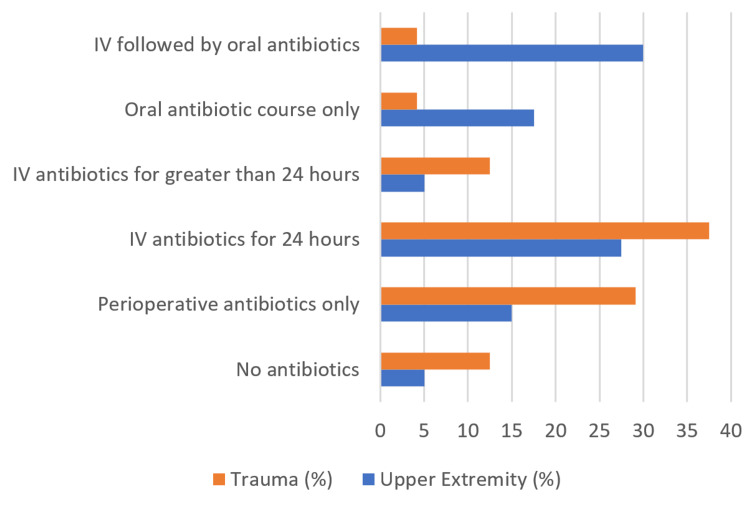
Postoperative antibiotic protocol after surgical fixation of grade 1 open distal radius fractures

Of the surgeons, 48% administered antibiotics for a total of 24 hours, while 45% of surgeons administered antibiotics for greater than 24 hours (Figure 6).

Of the surgeons, 65% stated that they would realistically perform an irrigation and debridement within 24 hours of the time of injury presentation. A total of 90% of surgeons stated that the presence of an open wound did not influence their surgical fixation choice (if otherwise indicated). In a theoretical situation of a patient with a grade 1 open distal radius fracture who presented greater than 72 hours after injury, with repeat X-rays demonstrating stable and acceptable alignment, 90% of surgeons would treat the fracture nonoperatively. A total of 68% of surgeons stated that litigation was not a major factor of concern.

## Discussion

Despite emerging evidence that conservative management is a reasonable option for certain open fractures, the historic standard of care for open fractures of any injury severity remains emergent systemic antibiotic administration, followed by urgent irrigation and debridement in the operating room. Our data suggest some practicing surgeons deviate from the historically established standard of care. While the polled surgeons practiced in a variety of settings, the majority of surgeons completed a fellowship in either orthopedic trauma or hand/upper extremity (see Figures 1, 2).

Many factors influence surgeon decision-making processes beyond the simple binary presence of an open wound. Regarding proper treatment regimen, surgeons considered factors such as level of contamination, fracture pattern, displacement on follow-up, and patient comorbidities (Figure 3). This finding contradicts the historical model that any open distal radius fracture de facto requires emergent irrigation and debridement in the operating room. Considering the isolated presence of a grade 1 open wound, 90% of polled surgeons would not change their surgical fixation choice if surgery were indicated.

Respondents noted several treatment algorithms for grade 1 open distal radius fractures as reasonable, ranging from entirely nonoperative management to emergent open reduction and internal fixation. When compared to trauma surgeons, more hand/upper extremity trained surgeons were willing to consider entirely nonoperative management with maintained acceptable fracture alignment after closed reduction (p = 0.024, Figure 4). While lacking good evidence in adults, conservative management of these injuries is potentially parallel to strong evidence suggesting that nonoperative treatment of grade 1 open forearm fractures in pediatric patients does not lead to an infection risk [2,11,12]. In a theoretical situation of a patient with a grade 1 open distal radius fracture who presented greater than 72 hours after injury, with the repeat X-rays demonstrating stable and acceptable alignment, 90% of surgeons would treat the fracture nonoperatively. The “standard of care” model appears to lag behind practicing surgeons.

In contrast to the above findings, some surgeons consider reasonable management to be more aggressive than the historical standard of care guidelines with regard to antibiotic duration. Whereas all surgeons administered intravenous (IV) antibiotics, trauma surgeons followed historical guidelines using only IV antibiotics. Upper extremity-trained surgeons were more willing to consider oral antibiotics following IV antibiotics (p < 0.001). Surgeons overwhelmingly (92%) administered cephalosporin antibiotics alone assuming no patient allergies. Interestingly, despite good evidence that antibiotic administration beyond 24 hours after irrigation and debridement for grade 1 fractures increases complications without additional benefit, 45% of surgeons administered antibiotics for greater than 24 hours (Figure 6).

No conclusive guidelines exist for the appropriate timing, or even necessity, of irrigation and debridement performed in the operating room for grade 1 open distal radius fractures. The historical evidence, dictating any open fracture requires irrigation and debridement within six hours, was promoted through mysterious origins, possibly from Friedrich in 1898 or Robson in 1973. More recently, numerous studies have questioned this “six-hour rule” [13-16]. While Schenker et al. did not fully reject the “six-hour rule,” they performed a meta-analysis that failed to demonstrate an association between early or delayed debridement and increased infection rate considering all infections, deep infections only, or severity of open fracture injuries [17]. Furthermore, some authors suggest operative irrigation and debridement may not be necessary for low-grade fractures at all [11]. Yang et al. demonstrated a 0% infection risk for 91 patients with isolated grade 1 open fractures managed by local wound care (without debridement) and IV antibiotics started within six hours and then continued for 48 hours from the time of injury [11].

As a pilot study, this study has important limitations. First, while we were able to display statistical significance for a portion of the survey responses, our relatively small sample size lacked the power to fully evaluate all of the survey responses. Second, surgeons reported many answers regarding reasonable treatment options when asked how they might definitely treat grade 1 open distal radius fractures. In future studies, we would like to pose targeted clinical scenarios with patient presentations, clinical photographs, and X-rays, so respondents can directly answer how they would treat specific grade 1 open distal radius fractures. Based on our results, further studies should examine the retrospective or prospective analysis of nonoperative management of grade 1 open distal radius fractures in adults.

Our data suggest some practicing surgeons deviate from the historically established standard of care. Some surgeons considered entirely nonoperative management of grade 1 open distal radius fractures, which has scant supporting evidence in adults. Some surgeons used methods of practice that were far more conservative than the current body of literature supports, such as duration of antibiotic use. To optimize the standard of care treatment guidelines, we need further studies examining outcomes of nonoperative management of grade 1 open distal radius fractures in adults.

## Conclusions

Standard of care management for all open fractures historically mandates emergent systemic antibiotic administration, followed by urgent irrigation and debridement in the operating room. Our data suggest some surgeons who manage grade 1 open distal radius fractures are willing to consider less aggressive treatment protocols as reasonable alternatives, potentially avoiding operative treatment entirely. Furthermore, when compared to surgeons who trained in a trauma fellowship, surgeons who trained in a hand/upper extremity fellowship are more willing to consider oral antibiotics as a supplement to IV antibiotics and management without open reduction and internal fixation entirely. Our pilot study highlights differences between the standard of care and modern practices.

## Appendices

Survey

Northwell Health Orthopaedic Institute

Management of grade 1 open distal radius fractures

Please share your feedback with us:

1. What type of residency program did you complete?

a) Orthopedic surgery

b) Plastic surgery

c) General surgery

2. Did you train in a fellowship program?

a) No

b) Upper extremity

c) Trauma

d) Sports

e) Oncology

f) Joint reconstruction

g) Spine

h) Pediatrics

i) Foot and ankle

3. What setting do you predominantly practice in?

a) Urban, academic

b) Suburban, academic

c) Urban, community

d) Suburban, community

e) Rural, community

4. Excluding antibiotic administration and debridement considerations, what is a reasonable definitive treatment protocol for a displaced, Gustillo-Anderson grade 1 open distal radius fracture? Select all applicable responses.

a) Closed reduction alone if alignment remains acceptable on follow-up

b) Closed reduction followed by emergent (less than eight hours) open reduction and internal fixation

c) Closed reduction followed by urgent (less than 48 hours) open reduction and internal fixation

d) Closed reduction followed by delayed (greater than 48 hours) open reduction and internal fixation

e) Closed reduction followed by closed reduction and percutaneous pinning

f) Closed reduction followed by external fixation

g) Closed reduction followed by operative management if loss of acceptable reduction

h) Open reduction and internal fixation alone

i) External fixation alone

j) Other (please specify)

5. What criteria do you use to determine your treatment regimen for grade 1 open distal radius fractures? Select all applicable responses.

a) Level of contamination

b) Mechanism of injury

c) Fracture pattern

d) Fracture displacement on follow up

e) Age of the patient

f) Comorbidities of the patient

6. At the time of injury, how would you address the patient’s tetanus immunization status?

a) Do not address tetanus status

b) Provide tetanus prophylaxis/treatment, regardless of patient’s immunization status

c) Provide tetanus prophylaxis/treatment as recommended by current Center for Disease Control Guidelines

d) Other (please specify)

7. Do you prescribe antibiotics at the time of injury?

a) No

b) Yes, IV antibiotics only

c) Yes, IV antibiotics followed by oral antibiotics

d) Yes, oral antibiotics only

8. Assuming a clean-contaminated grade 1 open distal radius fracture and that the patient had no allergies, what antibiotics would you prescribe?

a) Cephalosporin

b) Cephalosporin with aminoglycoside

c) Penicillin in addition to another antibiotic

d) Other (please specify)

9. Regardless of surgical intervention, if prescribing oral (PO) or IV antibiotics for grade 1 open distal radius fractures, what is the duration of antibiotic treatment?

a) PO antibiotics only, 24 hours

b) PO antibiotics only, greater than 24 hours

c) IV antibiotics followed by PO antibiotics, 24 hours combined

d) IV antibiotics followed by PO antibiotics, greater than 24 hours combined

10. Where do you first irrigate and debride the wound? Would you close the wound?

a) Do not irrigate and debride, do not close

b) Do not irrigate and debride, close primarily in the ED

c) Do not irrigate and debride, close primarily in the OR

d) In the ED and close primarily

e) In the ED but do not close

f) In the OR and close primarily

g) In the OR but do not close

11. If surgically treating a grade 1 open distal radius fracture with internal fixation, would you primarily close the injury wound or leave it open?

a) Close the wound

b) Do not close the wound

c) Incorporate wound into approach incision and close

12. What is the realistic time at which you would perform a debridement in the operating room?

a) Not applicable

b) Within six hours

c) Within 24 hours

d) Within 72 hours

e) Within one week

f) Greater than one week

13. Does your surgical fixation choice differ between a grade 1 open distal radius fracture and a closed distal radius fracture?

a) Yes

b) No

14. In relation to the administration of systemic antibiotics, what role do you believe local antibiotics have in grade 1 open distal radius fractures?

a) Local antibiotics have no role

b) Local antibiotics have an adjuvant role

c) Local antibiotics may be used instead of systemic antibiotics

15. If you perform surgical fixation of the fracture, what is your postoperative antibiotic protocol?

a) No antibiotics

b) Perioperative antibiotics only

c) IV antibiotics for 24 hours

d) IV antibiotics for greater than 24 hours

e) Oral antibiotic course only

f) IV followed by oral antibiotics

16. If a patient with a grade 1 open distal radius fracture presented greater than 72 hours after injury with repeat X-rays demonstrating stable and acceptable alignment, would you treat the fracture nonoperatively?

a) Yes

b) No

17. If an elderly patient with insignificant medical comorbidities and good functional status presented with a displaced grade 1 open distal radius fracture, would the presence of an open fracture necessitate operative irrigation and debridement?

a) Yes

b) No

18. Is concern for litigation a major factor in your treatment algorithm for grade 1 open distal radius fracture?

a) Yes

b) No
